# The Th17/Treg Cytokine Imbalance in Chronic Obstructive Pulmonary Disease Exacerbation in an Animal Model of Cigarette Smoke Exposure and Lipopolysaccharide Challenge Association

**DOI:** 10.1038/s41598-019-38600-z

**Published:** 2019-02-13

**Authors:** Daniela A. B. Cervilha, Juliana T. Ito, Juliana D. Lourenço, Clarice R. Olivo, Beatriz M. Saraiva-Romanholo, Rildo A. Volpini, Manoel C. Oliveira-Junior, Thais Mauad, Milton A. Martins, Iolanda F. L. C. Tibério, Rodolfo P. Vieira, Fernanda D. T. Q. S. Lopes

**Affiliations:** 1 Department of Medicine, Laboratory of Experimental Therapeutics (LIM-20), School of Medicine, University of Sao Paulo, Sao Paulo, Brazil; 2 Department of post-graduation of Institute of Medical Assistance to the State Public Servant, University City of Sao Paulo, Sao Paulo, Brazil; 3 Nephrology Department, School of Medicine, University of Sao Paulo, Sao Paulo, Brazil; 4 Laboratory of Pulmonary and Exercise Immunology, Nove de Julho University, Sao Paulo, Brazil; 5 Department of Pathology, School of Medicine, University of Sao Paulo, Sao Paulo, Brazil; 6 Post-graduation Program in Bioengineering and in Biomedical Engineering, Universidade Brasil, Sao Paulo, Brazil; 7 Post-graduation Program in Sciences of Human Movement and Rehabilitation, Federal University of Sao Paulo (UNIFESP), Santos, Brazil; 8 Brazilian Institute of Teaching and Research in Pulmonary and Exercise Immunology (IBEPIPE), Sao Jose dos Campos, Brazil

## Abstract

We proposed an experimental model to verify the Th17/Treg cytokine imbalance in COPD exacerbation. Forty C57BL/6 mice were exposed to room air or cigarette smoke (CS) (12 ± 1 cigarettes, twice a day, 30 min/exposure and 5 days/week) and received saline (50 µl) or lipopolysaccharide (LPS) (1 mg/kg in 50 µl of saline) intratracheal instillations. We analyzed the mean linear intercept, epithelial thickness and inflammatory profiles of the bronchoalveolar lavage fluid and lungs. We evaluated macrophages, neutrophils, CD4^+^ and CD8^+^ T cells, Treg cells, and IL-10^+^ and IL-17^+^ cells, as well as STAT-3, STAT-5, phospho-STAT3 and phospho-STAT5 levels using immunohistochemistry and IL-17, IL-6, IL-10, INF-γ, CXCL1 and CXCL2 levels using ELISA. The study showed that CS exposure and LPS challenge increased the numbers of neutrophils, macrophages, and CD4^+^ and CD8^+^ T cells. Simultaneous exposure to CS/LPS intensified this response and lung parenchymal damage. The densities of Tregs and IL-17^+^ cells and levels of IL-17 and IL-6 were increased in both LPS groups, while IL-10 level was only increased in the Control/LPS group. The increased numbers of STAT-3, phospho-STAT3, STAT-5 and phospho-STAT5^+^ cells corroborated the increased numbers of IL-17^+^ and Treg cells. These findings point to simultaneous challenge with CS and LPS exacerbated the inflammatory response and induced diffuse structural changes in the alveolar parenchyma characterized by an increase in Th17 cytokine release. Although the Treg cell differentiation was observed, the lack of IL-10 expression and the decrease in the density of IL-10^+^ cells observed in the CS/LPS group suggest that a failure to release this cytokine plays a pivotal role in the exacerbated inflammatory response in this proposed model.

## Introduction

Chronic obstructive pulmonary disease (COPD) is the fourth highest cause of mortality in the world, and it is predicted to become the third cause of death worldwide by 2020^1,2^. The use of tobacco has been identified as a main risk factor for the development of this disease^3^.

After many years of smoking, the lungs become inflamed and exhibit the hypersecretion of mucus, providing a site for colonization of infectious pathogens^4^ that could culminate in the exacerbation of respiratory diseases^5–10^. Clinical studies have identified a correlation between recurrent respiratory bacterial^11^ or viral infections and COPD exacerbation^10,12–14^, and the pivotal roles of innate and adaptive immune responses in the worsening of this lung disease^15,16^.

Regarding the innate immune response, macrophages are part of the first line of lung defense in early events of infections induced by bacterial or viral agents. These cells phagocyte microbes and apoptotic cells to eliminate deleterious agents and also are responsible for releasing some pro-inflammatory mediators that promoteing neutrophils migration to the pulmonary site^17^. However, cigarette smoke (CS) exposure impairs macrophages activity^17^, and the persistence of this inflammatory process culminates in COPD progression^15,17^.

COPD progression is associated with an infiltration of CD8^+^ and CD4^+^ T lymphocytes mainly into the small airways^15,18^.

The effectors immune responses result from the differentiation of naïve CD4^+^ T cells into Th1, or Th2, or Th17 or regulatory T cells (Treg) depending on the cytokines that signal through the Janus kinase (JAK) - signal transducer and activator of transcription (STAT) pathway^19,20^.

Interleukin (IL)-6, IL-23 and tumor growth factor-beta (TGF-β) activate STAT3 and subsequently induce Th17 differentiation. In contrast, Treg differentiation depends on the presence of IL-12 and TGF-β to activate STAT5^21^.

Treg cells are recognized by their ability to suppress inflammation and to inhibit autoimmunity^22^. Anti-inflammatory cytokines such as IL-10 and TGF-β are also released by Treg cells^23,24^. In our previous study, a decrease in the numbers of Treg cells and TGF-β^+^ and IL-10^+^ cells was associated with an increase in the number of IL-17^+^ cells in the airways of smokers, leading to obstructions^25^.

Although the importance of Treg cells in COPD progression has been described^25^, the importance of this T cell subtype in COPD exacerbation remains unclear.

On the other hand, the Th17 response has been described both in COPD progression^26^ and in bacterial infections in patients with COPD presenting exacerbations. Ross and colleagues^27^ observed increased IL-17A levels in the bronchoalveolar lavage fluid (BALF) and lung tissues of patients with COPD, followed by neutrophil recruitment during acute exacerbations induced by a *Haemophilus influenzae* infection.

Lipopolysaccharide (LPS) is a pro-inflammatory component of gram-negative bacteria that is present in high amounts in CS^28–30^. It has been extensively used in animal models to induce systemic inflammation and, depending on the dose, is capable of inducing pulmonary emphysema^31^.

Recently, LPS has been used in murine models to resemble COPD exacerbations in humans. Kobayashi and colleagues^32^ proposed a model of COPD exacerbation combining the instillation of elastase and LPS and verified an infiltration of CD8^+^ T cells into alveolar spaces and an increase in metalloproteinase-9 and perforin levels in the BALF. Additionally, Vernooy and colleagues^33^ observed chronic lung inflammation characterized by the presence of lymphocytic aggregates in peribronchial and perivascular areas following long-term exposure to LPS.

Since tobacco smoking is the main etiological factor contributing to the development of COPD in humans and bacterial infections are known to induce an adaptive immune response resulting in the exacerbation of this disease, in this present study, we intend to verify the role of the adaptive immune response in COPD exacerbation using a CS exposure model treated with an LPS instillation, focusing on the Th17/Treg cytokine imbalance in this process.

## Material and Methods

### Animals

Forty male C57BL/6 mice (20–25 g), aged 6–8 weeks were used in this study. This study was performed using a protocol approved by the Ethics in Research Committee for Human and Animal Studies of University of São Paulo School of Medicine (São Paulo, Brazil) (protocol number 077/14). All animals received humane care in compliance with the Guide for the Care and Use of Laboratory Animals published by the US National Institutes of Health (NIH Publication N°. 85–23, revised 1996).

### Experimental groups

**Figure 1:**
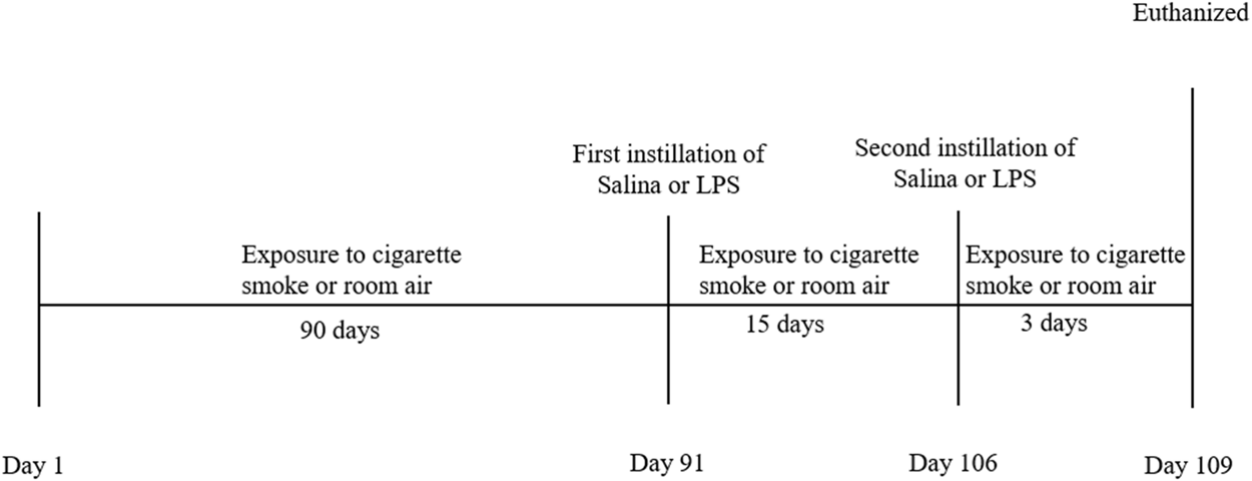
Timeline of the experimental protocol.

Mice were either exposed to room air and received two instillations of a saline solution (Control/SAL) or LPS solution (Control/LPS), or were exposed to CS and received two instillations of a saline solution (CS/SAL) or LPS solution (CS/LPS) (Fig. 1).

### Experimental protocol

Animals were exposed to CS or room air for 3 months. They received saline or LPS instillations on the 91^st^ and 106^th^ days. Animals were euthanized three days after the last instillation (Fig. 1).

### Induction of emphysema

Animals were exposed to CS using methods previously described by Toledo and colleagues to induce COPD^34^. We used a plastic box (28 L) with two inlets for synthetic air and smoke supplies. An airflow of 2 L/min was maintained and regulated by a flowmeter that passed through a Venturi System connected to a lit cigarette, providing the suction of CS into the box. A flow rate was set to produce carbon monoxide (CO) levels ranging from 250 to 350 ppm. Approximately 12 (±1) commercially filtered cigarettes were used per day (0.8 mg of nicotine, 10 mg of tar and 10 mg of CO per cigarette), with a total particulate matter concentration of 354.8 ± 50.3 μg/m^3^/day. The animals were housed in the smoke environment for 30 min/day, 2 times/day for 5 days/week for a period of 14 weeks. The control groups remained in room air (Fig. 1).

### Intratracheal LPS challenge

The intratracheal challenge with LPS (*E*. *coli*, serotype O26:B6: Sigma Chemical Co.) was performed to simulate bacterial stimuli via an anterior cervical incision to expose the trachea. Animals received the LPS (1 mg/kg diluted in 50 µl of a sterile saline solution) or sterile saline solution (0.9% NaCl; 50 µl)^32^. Mice were placed on a heated animal bed (30 °C) until they showed active movements. They were also subjected to post-surgical recovery procedure are received tramadol chlorhydrate (5 mg/mL) via intramuscular injections (Fig. 1). Thus, we did not observe any considerable differences in animal’s behavior. After the recovery period, animals were returned to the cage under normal conditions. The animals were active and did not display signs of post-operative pain.

### Bronchoalveolar lavage fluid (BALF)

At the end of the protocol, mice were intraperitoneally anaesthetized with thiopental (70 mg/kg) and euthanized by transecting the abdominal aorta. BALF was obtained through the tracheal cannula by washing the airway lumen with 3 × 0.5 ml of sterile saline. BALF samples were centrifuged at 1000 rpm at 4 °C for 10 min and the cell pellet was resuspended in 0.2 mL of sterile saline for the Control groups and 1.5 mL of sterile saline for LPS groups. Total cell numbers were determined using a Neubauer hemocytometer counting chamber (Carl Roth, Karlsruhe, Germany). Differential cell counts were evaluated by a microscopic examination of BALF samples prepared on cytocentrifuge slides that were stained with Diff Quick (Medion Diagnostics, Dündingen, Switzerland)^35^.

### Lung preparation

Lungs were removed en bloc and fixed with 4% formaldehyde infused through the trachea at constant pressure of 20 cmH_2_O for 24 h. Lungs were embedded in paraffin and cut into 5 µm coronal sections.

### Morphometry

For conventional morphometry, we used an eyepiece with a coherent system of 50 lines and 100 points with a known area attached to the microscope ocular to perform the mean linear intercept (Lm) measurements, an indicator of the mean alveolar diameter^36^. Tissue samples were stained with hematoxylin and eosin (H&E). For each animal, images of 20 fields at a magnification of 200× were captured. Lm was obtained by counting the number of times that the lines of the reticulum intercepted the alveolar walls and calculated using the following equation: Lm = Ltotal/NI.

We performed the Lm analysis in subpleural airspaces and peribronchial airspaces.

### Epithelial thickness

Histological sections were stained with H&E to evaluate the epithelial thickness. We used the Panoramic Viewer 1.5 (3DHISTECH, Budapest, Hungary), an image analysis system. We quantified 5 airways from each animal at a magnification of 400×. Epithelial thickness was defined as the distance between the basement membrane and the luminal cell membrane, excluding the cilia^37^, and the length of basement membrane was determined. The epithelial thickness was expressed as a relationship between the epithelial thickness and the length of basement membrane.

### Immunohistochemistry

Tissue sections were deparaffinized and hydrated. After blocking endogenous peroxidase activity, antigen retrieval was performed with a high-temperature citrate buffer (pH = 6.0). The primary antibodies used in this study were: a rat monoclonal antibody against Mac-2 (1:50000, Cedarlane, Ontario, CA), a rabbit polyclonal antibody against neutrophil elastase (1:2000, Abcam, Cambridge, UK), a rabbit polyclonal antibody against FOXP3 (1:500, Abcam, Cambridge, UK), a rat monoclonal antibody against CD4 (1:10, Santa Cruz, CA, USA), a rabbit polyclonal antibody against CD8 (1:100, Abcam, Cambridge, UK), a rabbit polyclonal antibody against IL-17 (1:200, Abcam, Cambridge, UK), and a rat monoclonal antibody against IL-10 (1:30, Santa Cruz, CA, USA), a rabbit polyclonal antibody against STAT3 (1:1000, Santa Cruz, CA, USA), a goat polyclonal antibody against phospho-STAT3 (1:2000, Santa Cruz, CA, USA), a goat polyclonal antibody against STAT5 (1:2000, Santa Cruz, CA, USA), and a goat polyclonal antibody against phospho-STAT5 (1:100, Santa Cruz, CA, USA). The Vectastain ABC Kit (Vector Laboratories, Burlingame, CA, USA) was used in conjunction with a species-specific secondary antibody or Histofine Polymer (Nichirei Biosciences, Tokyo, JP), and the sections were stained using chromogen diaminobenzidin (DAB, Sigma, St. Louis, MO, USA). Sections were counterstained with Harris hematoxylin (Merck, Darmstadt, Germany). For negative controls, the primary antibody was omitted^34^ and replaced with BSA for the incubation with tissue sections.

Images of the lung tissues were captured from 15–20 random parenchymal fields for each lung sample at 400× magnification using the Panoramic Viewer 1.5 (3DHISTECH, Budapest, Hungary). We used a digital analysis system and specific software (Image-Pro Plus version 4.5 for Windows, Media Cybernetics, MD, USA) to determine the area of pulmonary parenchyma. Then, we quantified the density of positive cells for Mac-2, neutrophil elastase, CD4, CD8, FOXP3, IL-10, IL-17, STAT3, phospho-STAT3, STAT5 and phospho-STAT5 per parenchymal area, which was reported as cells/μm^2^.

### Double immunohistochemical staining

We performed double immunostaining to examine the deficiency in IL-10 expression in Treg cells. Firstly, lung tissue sections were stained with a rabbit polyclonal against FOXP3 (1:300, Abcam, Cambridge, UK) using an immunoperoxidase procedure and DAB as chromogen. Afterwards, sections were incubated with a rat monoclonal antibody against IL-10 (1:100, Santa Cruz, CA, USA) using an equivalent protocol with an immunoalkaline phosphatase procedure and a red colored reaction product (PermaRed/AP, Diagnostic BioSystems, Pleasanton, CA). Finally, Harris hematoxylin was used to counterstain tissue sections. Images of the lung tissues were captured with an AxioCam digital camera MRc5 using the software Zen from Carl Zeiss (München-Hallbergmoos, Germany). We captured images at magnifications of 400× and 1000×.

### Cytokine analysis

Lungs were homogenized in a saline solution (0.9% NaCl), centrifuged, and the supernatants were stored at −80 °C until subsequent analyses. The levels of IL-17, interferon-gamma (IFN-γ), chemokine C-X-C motif ligand 1 (CXCL1) and CXCL2 were quantified using enzyme linked immunosorbent assays (ELISA). The kits were purchased from R&D System (Minneapolis, MN), and the levels of IL-6 and IL-10 were quantified using ELISA kits purchased from BioLegend (San Diego, CA). The ELISA was performed according to the manufacturers’ instructions.

### Statistical analysis

The statistical analysis was performed using SigmaStat statistical software (Systat Software, San Jose, CA). For immunohistochemistry and cytokine analyses, we logarithmically transformed the data. Data were analyzed using one-way ANOVA followed by Holm-Sidak post hoc analysis for data with a parametric distribution or Kruskal-Wallis test for data with a nonparametric distribution and Dunn’s post hoc analysis. Results are presented as means ± SE and p value < 0.05 was considered statically significant.

### Ethical approval

This study was performed under a protocol approved by the Ethics in Research Committee for human and animal studies of University of São Paulo School of Medicine (São Paulo, Brazil) (protocol number 077/14). All animals received humane care in compliance with the Guide for the Care and Use of Laboratory Animals published by the US National Institutes of Health (NIH Publication N°. 85–23, revised 1996).

## Results

### Inflammatory changes in BALF

**Figure 2:**
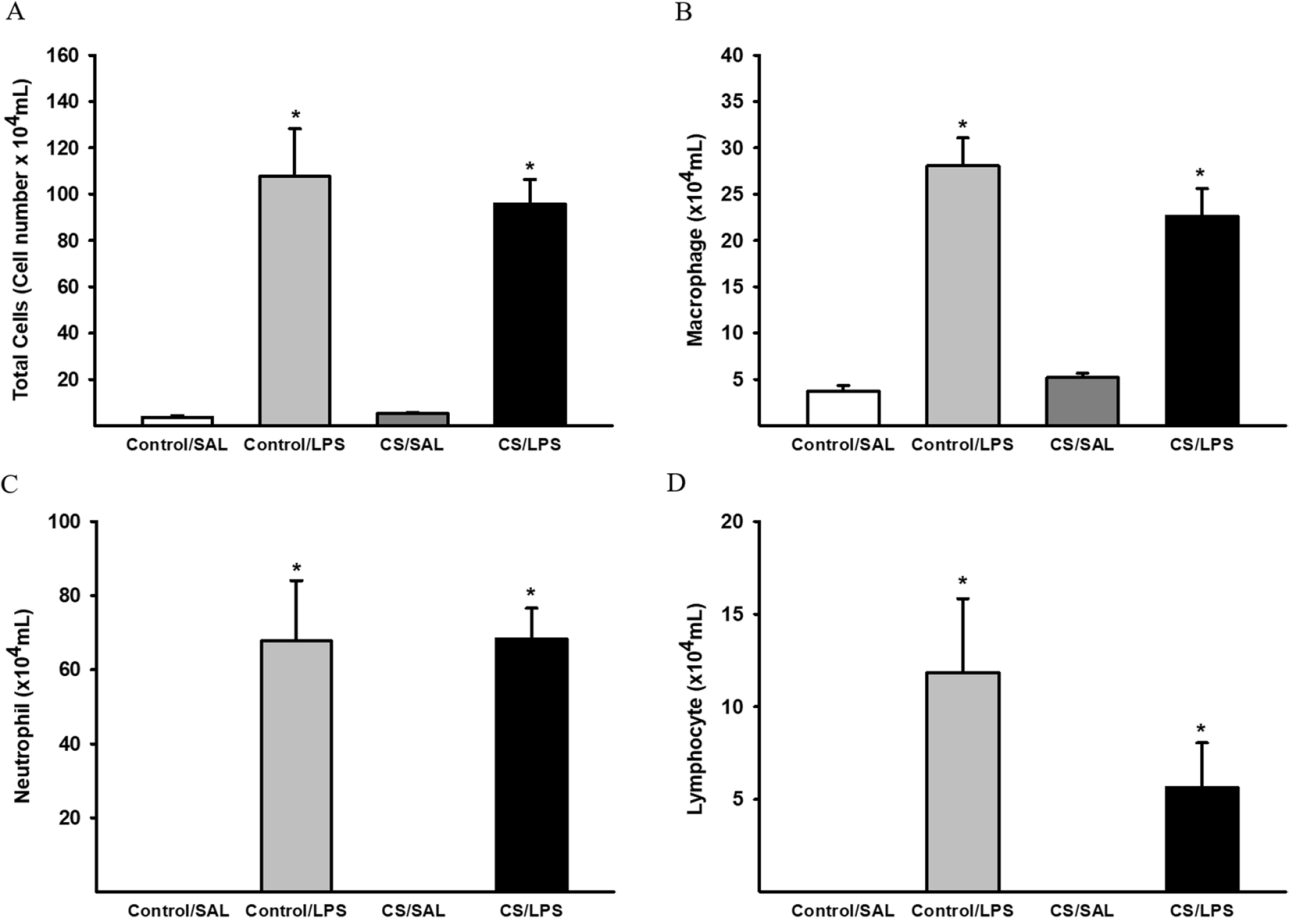
Inflammatory cells in the BAL. (**A**) Total cells, *p < 0.001 compared to Control/SAL and CS/SAL; Control/SAL n = 10, Control/LPS n = 6, CS/SAL n = 9, CS/LPS n = 7. ANOVA, Holm-Sidak post hoc. (**B**) Macrophages, *p < 0.001 compared to Control/SAL and CS/SAL; Control/SAL n = 10, Control/LPS n = 7, CS/SAL n = 6, CS/LPS n = 7. (**C**) Neutrophils, *p < 0.001 compared to Control/SAL and CS/SAL; Control/SAL n = 10, Control/LPS n = 6, CS/SAL n = 7, CS/LPS n = 7. (**D**) Lymphocyte, *p < 0.001 compared to Control/SAL and CS/SAL. Control/SAL n = 10, Control/LPS n = 4, CS/SAL n = 7, CS/LPS n = 7. Kruskal-Wallis, Dunn’s post hoc. Data are presented as means and SE.

The intratracheal instillation of LPS induced a significant increase in the numbers of total cells, macrophages, neutrophils and lymphocytes in the BALF compared to the Control/SAL and CS/SAL groups (Fig. 2A–D).

### Lung morphometry

**Figure 3:**
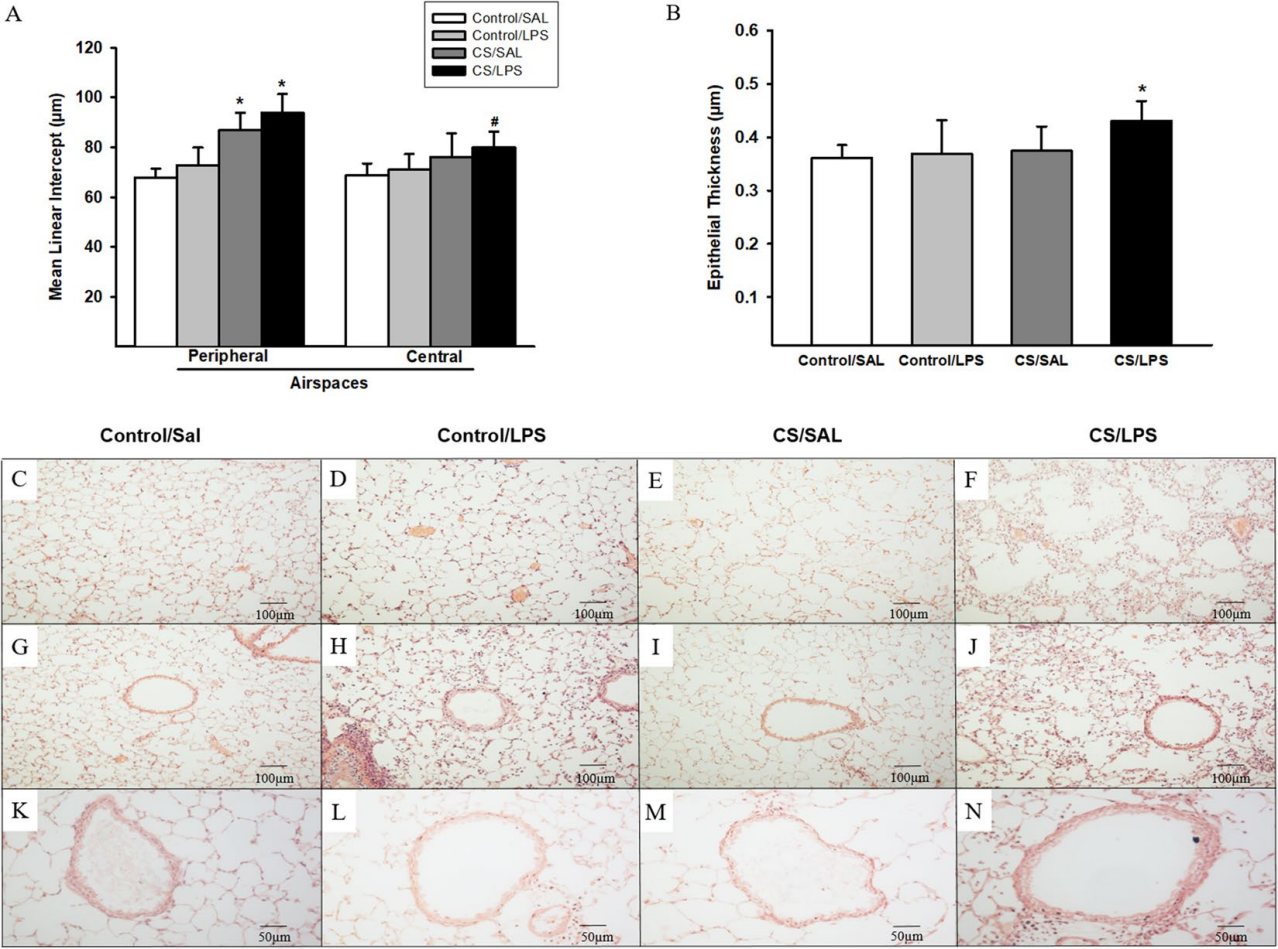
Mean linear intercept measured in subpleural airspaces and peribronchial airspaces and epithelial thickness in the experimental groups. (**A**) Lm: *p < 0.001 compared to Control/SAL and Control/LPS groups. Control/SAL n = 9, Control/LPS n = 9, CS/SAL n = 8, CS/LPS n = 5. ANOVA, Holm-Sidak post hoc. ^#^p = 0.02 compared to Control/SAL. Control/SAL n = 9, Control/LPS n = 9, CS/SAL n = 8, CS/LPS n = 5. Kruskal-Wallis, Dunn’s post hoc. Photomicrographs of Lm in subpleural airspaces and peribronchial airspaces. (**C**,**G**) Control/SAL group; (**D**,**H**) Control/LPS group; (**E**,**I**) CS/SAL group and (**F**,**J**) CS/LPS group, respectively, 200X magnification. (**B**) Epithelial thickness: *p = 0.02 compared to Control/SAL. Control/SAL n = 9, Control/LPS n = 9, CS/SAL n = 8, CS/LPS n = 5. ANOVA, Holm-Sidak post hoc. Photomicrographs of epithelial thickness in airways (**K**) Control/SAL group; (**L**) Control/LPS group; (**M**) CS/SAL group and (**N**) CS/LPS group. Data are presented as means and SE. 400X magnification.

Alveolar enlargement was observed in the subpleural airspaces of animals exposed to CS compared to Control groups. However, we only observed alveolar enlargement in the peribronchial airspaces in the CS/LPS group, but not the Control/SAL group (Fig. 3A,C–J).

An increase in epithelial thickness was observed in the CS/LPS group compared to the Control/SAL group (Fig. 3B,K–N).

### Lung tissue inflammation

**Figure 4:**
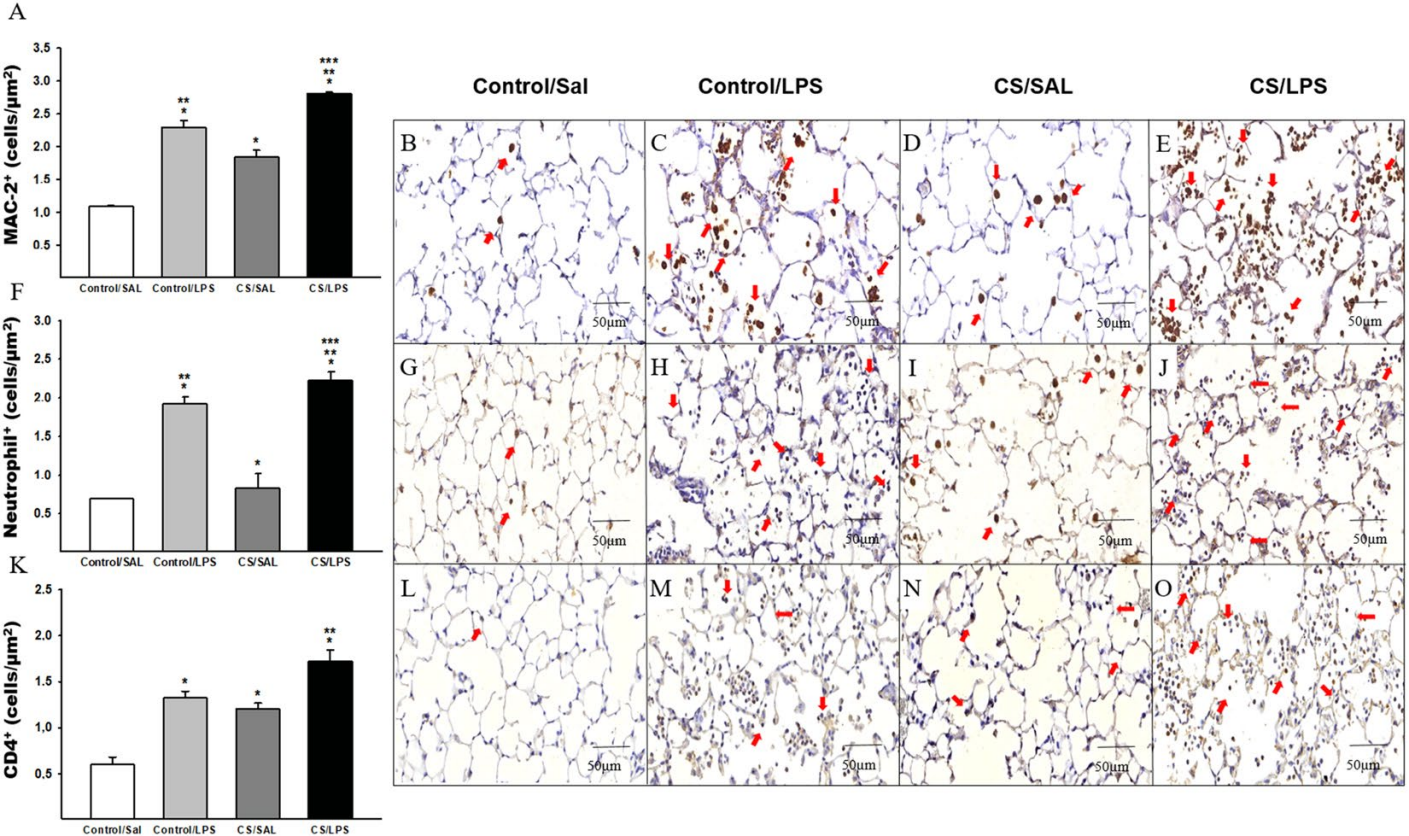
The density of positive cells for MAC-2, neutrophils and CD4^+^ in experimental groups. (**A**) MAC-2: *p < 0.001 compared to Control/SAL; **p < 0.001 compared to CS/SAL group and ***p < 0.001 compared to Control/LPS group. Control/SAL n = 9, Control/LPS n = 9, CS/SAL n = 7, CS/LPS n = 5. Photomicrographs of MAC-2 in lung parenchyma (**B**) Control/SAL group; (**C**) Control/LPS group; (**D**) CS/SAL group and (**E**) CS/LPS group, 400X magnification. (**F**) Neutrophils: *p < 0.001 compared to Control/SAL; **p < 0.001 compared to CS/SAL group and ***p < 0.001 compared to Control/LPS group. Control/SAL n = 9, Control/LPS n = 9, CS/SAL n = 8, CS/LPS n = 5. Photomicrographs of neutrophils in lung parenchyma (**G**) Control/SAL group; (**H**) Control/LPS group; (**I**) CS/SAL group and (**J**) CS/LPS group, 400X magnification. (**K**) CD4^+^: *p < 0.001 compared to Control/SAL. **p < 0.001 compared to CS/SAL and Control/LPS groups. Control/SAL n = 9, Control/LPS n = 9, CS/SAL n = 8, CS/LPS n = 5. Photomicrographs of CD4 T cells in lung parenchyma (**L**) Control/SAL group; (**M**) Control/LPS group; (**N**) CS/SAL group and (**O**) CS/LPS group, 400X magnification. Data were expressed as log_10_ base. ANOVA, Holm-Sidak post hoc. Data are presented as means and SE.

We observed increased densities of macrophages and neutrophils in the lung parenchyma of animals that were challenged with LPS compared to the Control/SAL and CS/SAL. We also observed an increase in the densities of these cells in the CS/LPS group compared to the Control/LPS group. Furthermore, we observed an increase in the CS/SAL group compared to the Control/SAL group (Fig. 4A–J, respectively).

**Figure 5:**
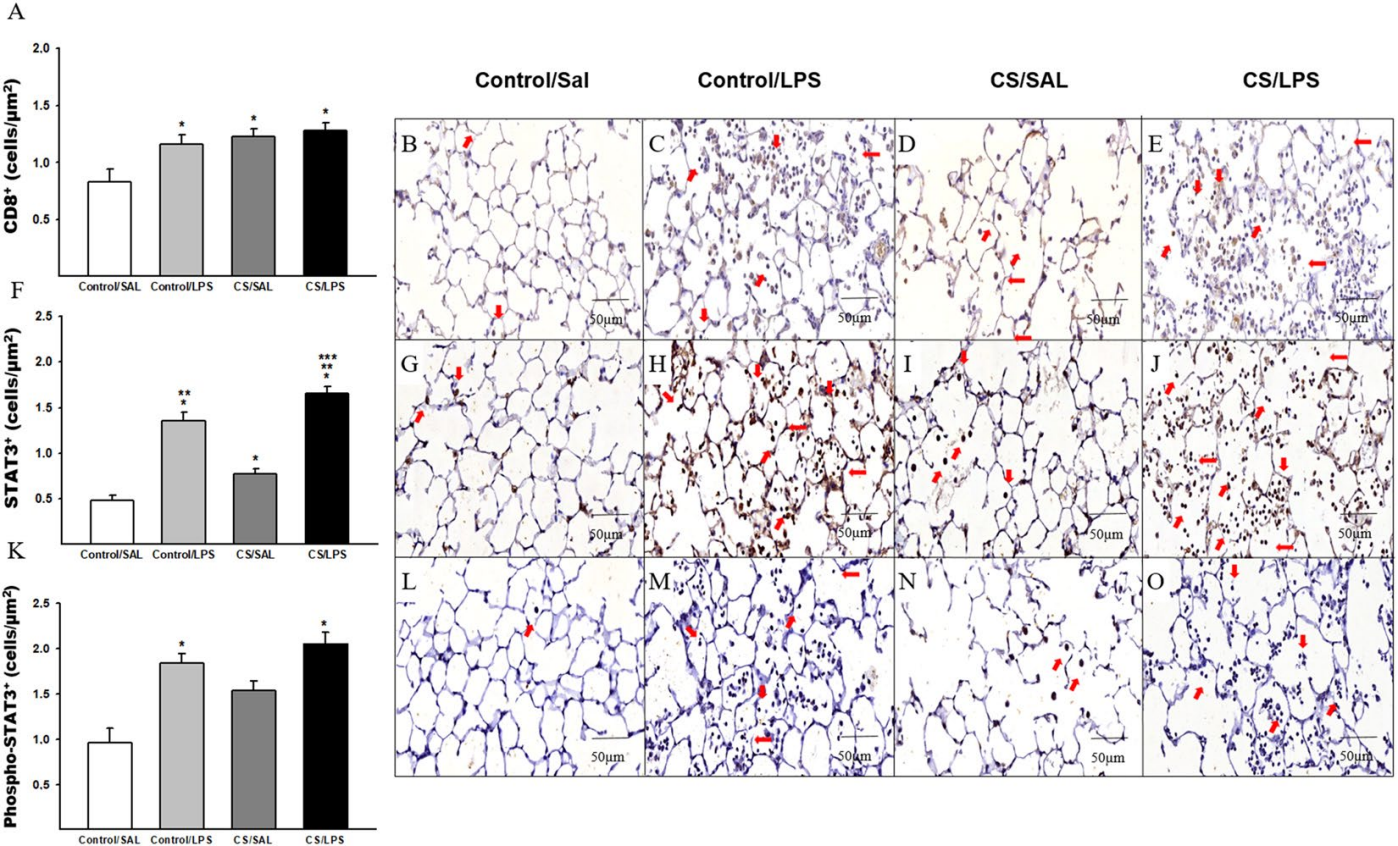
The density of positive cells for CD8^+^, STAT3 and Phospho-STAT3 in experimental groups. (**A**) CD8^+^: *p = 0.006 compared to Control/SAL. Control/SAL n = 9, Control/LPS n = 9, CS/SAL n = 8, CS/LPS n = 5. Photomicrographs of CD8 T cells in lung parenchyma (**B**) Control/SAL group; (**C**) Control/LPS group; (**D**) CS/SAL group and (**E**) CS/LPS group, 400X magnification. (**F**) STAT3: *p < 0.001 compared to Control/SAL; **p < 0.001 compared to CS/SAL group and ***p < 0.001 compared to Control/LPS group. Control/SAL n = 7, Control/LPS n = 9, CS/SAL n = 6, CS/LPS n = 5. Photomicrographs of STAT3 in lung parenchyma (**G**) Control/SAL group; (**H**) Control/LPS group; (**I**) CS/SAL group and (**J**) CS/LPS group, 400X magnification. Data were expressed as log_10_ base. ANOVA, Holm-Sidak post hoc. Data are presented as means and SE. (**K**) Phospho-STAT3: *p < 0.001 compared to Control/SAL. Control/SAL n = 8, Control/LPS n = 8, CS/SAL n = 8, CS/LPS n = 4. Photomicrographs of Phospho-STAT3 in lung parenchyma (**L**) Control/SAL group; (**M**) Control/LPS group; (**N**) CS/SAL group and (**O**) CS/LPS group, 400X magnification. Data were expressed as log_10_ base. Kruskal-Wallis, Dunn’s post hoc. Data are presented as means and SE.

The groups that received LPS challenge showed increased densities of CD4^+^ and CD8^+^ T cells compared to the Control/SAL group (Figs 4K–O and 5A–E, respectively). We also identified an increase in the CS/SAL group compared to the Control/SAL group. In addition, we observed an increase in the density of CD4^+^ cells in the CS/LPS group compared to the CS/SAL and Control/LPS groups(Fig. 4K–O).

**Figure 6:**
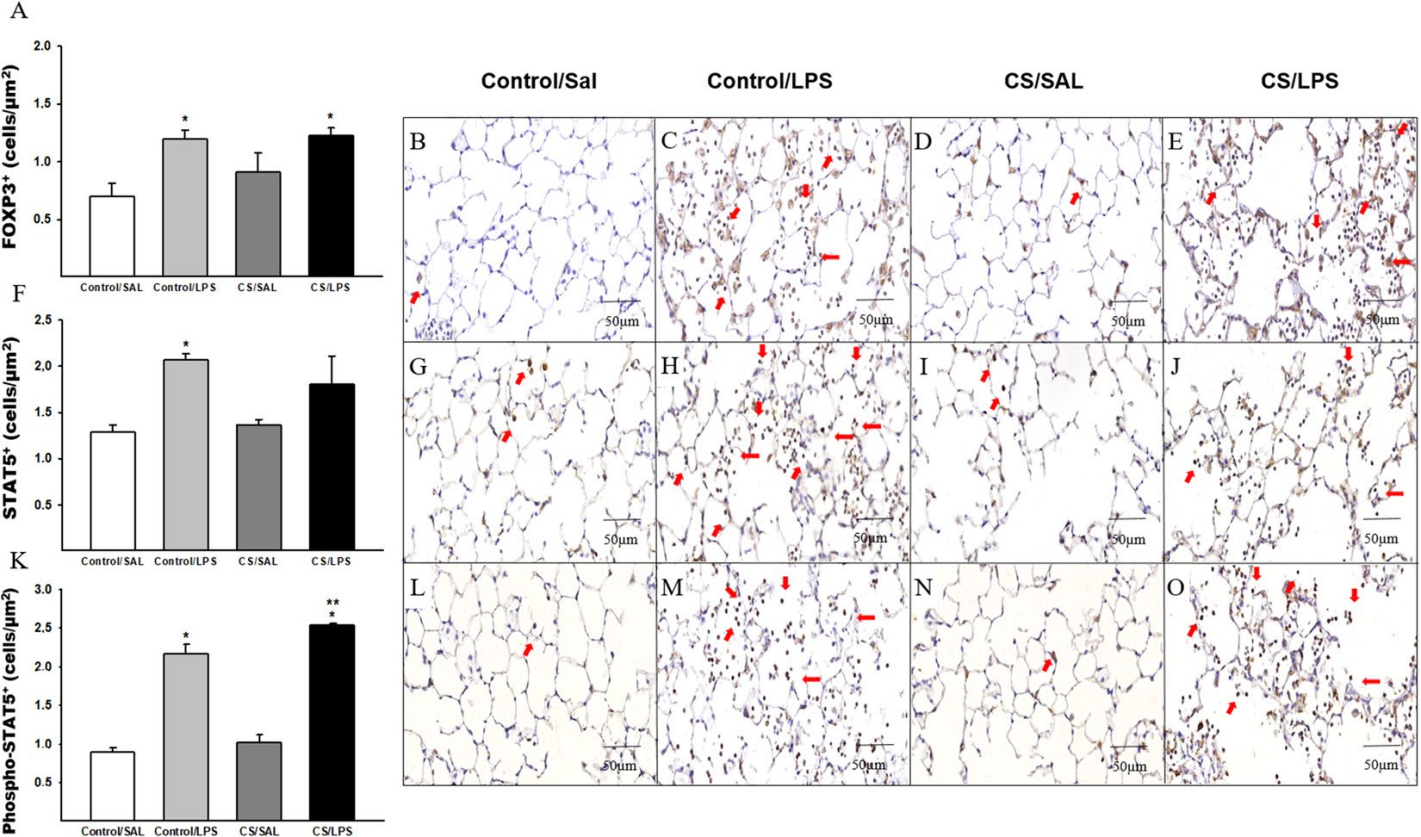
The density of positive cells for FOXP3, STAT5 and Phospho-STAT5 in experimental groups. (**A**) FOXP3: *p = 0.01 compared to Control/SAL. Control/SAL n = 9, Control/LPS n = 9, CS/SAL n = 8, CS/LPS n = 5. Photomicrographs of FOXP3 cells in lung parenchyma (**B**) Control/SAL group; (**C**) Control/LPS group; (**D**) CS/SAL group and (**E**) CS/LPS group, respectively, 400X magnification. Data were expressed as log_10_ base. ANOVA, Holm-Sidak post hoc. Data are presented as means and SE. (**F**) STAT5: *p < 0.001 compared to Control/SAL and CS/SAL groups. Control/SAL n = 9, Control/LPS n = 9, CS/SAL n = 8, CS/LPS n = 5. Photomicrographs of STAT5 in lung parenchyma (**G**) Control/SAL group; (**H**) Control/LPS group; (**I**) CS/SAL group and (**J**) CS/LPS group, 400X magnification. Data were expressed as log_10_ base. Kruskal-Wallis, Dunn’s post hoc. Data are presented as means and SE. (**K**) Phospho-STAT5: *p < 0.001 compared to Control/SAL and CS/SAL groups; **p < 0.001 compared to Control/LPS group. Control/SAL n = 9, Control/LPS n = 9, CS/SAL n = 8, CS/LPS n = 5. Photomicrographs of Phospho-STAT5 in lung parenchyma (**L**) Control/SAL group; (**M**) Control/LPS group; (**N**) CS/SAL group and (**O**) CS/LPS group, 400X magnification. Data were expressed as log_10_ base. ANOVA, Holm-Sidak post hoc. Data are presented as means and SE.

The LPS challenge (Control/LPS and CS/LPS) increased the density of FOXP3^+^ cells compared to Control/SAL group (Fig. 6A–E). The density of STAT3^+^ cells was increased in the lung parenchyma of animals challenged with LPS compared to the Control/SAL and CS/SAL groups. We also observed an increase in the CS/LPS group compared to the Control/LPS group. Furthermore, we observed an increase in the CS/SAL group compared to Control/SAL group (Fig. 5F–J). Moreover, the LPS challenge (Control/LPS and CS/LPS) increased the density of phospho-STAT3^+^ cells compared to the Control/SAL group (Fig. 5K–O). We observed an increased in the density of STAT5^+^ cells in the Control/LPS group compared to the Control/SAL and CS/SAL groups (Fig. 6F–J). In addition, we detected an increase in the density of phospho-STAT5^+^ cells in the lung parenchyma of animals challenged with LPS compared to the Control/SAL and CS/SAL groups, and we observed an increase in the CS/LPS group compared to Control/LPS group (Fig. 6K–O).

**Figure 7:**
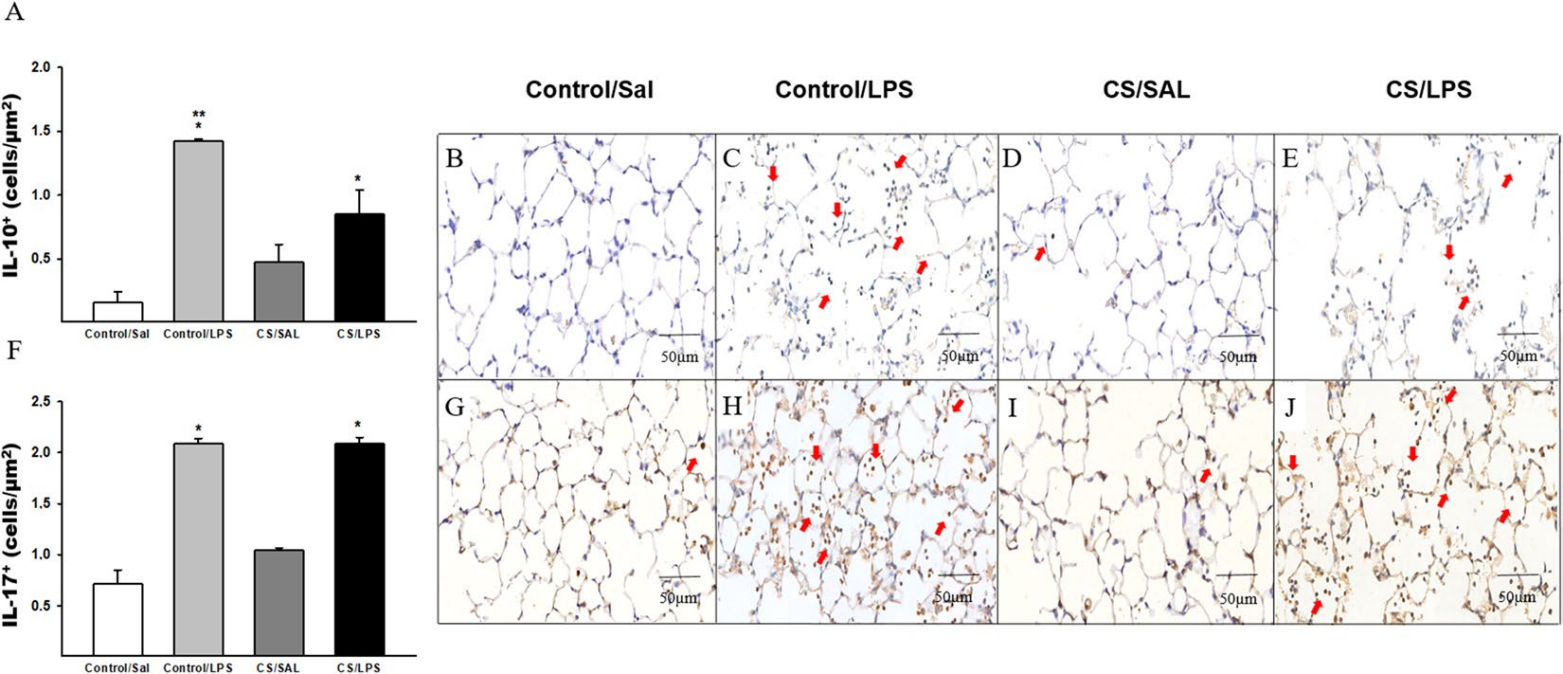
The density of positive cells for IL-10 and IL-17 in experimental groups. (**A**) IL-10: *p = 0.001 compared to Control/SAL; **p = 0.001 compared to CS/SAL and CS/LPS. Control/SAL n = 6, Control/LPS n = 6, CS/SAL n = 6, CS/LPS n = 5. Photomicrographs of IL-10 cells in lung parenchyma (**B**) Control/SAL group; (**C**) Control/LPS group; (**D**) CS/SAL group and (**E**) CS/LPS group, 400X magnification. Data were expressed as log_10_ base. ANOVA, Holm-Sidak post hoc. Data are presented as means and SE. (**F**) IL-17: *p < 0.001 compared to Control/SAL group. Control/SAL n = 6, Control/LPS n = 6, CS/SAL n = 6, CS/LPS n = 5. Photomicrographs of IL-17 in lung parenchyma (**G**) Control/SAL group; (**H**) Control/LPS group; (**I**) CS/SAL group and (**J**) CS/LPS group, 400X magnification. Data were expressed as log_10_ base. Kruskal-Wallis, Dunn’s post hoc. Data are presented as means and SE.

The density of IL-10^+^ cells was increased in the lung parenchyma of animals challenged with LPS compared to the Control/SAL group. We also observed an increase in the Control/LPS group compared to the CS/SAL and CS/LPS groups (Fig. 7A–E). Moreover, the LPS challenge (Control/LPS and CS/LPS) increased the density of IL-17^+^ cells compared to the Control/SAL group (Fig. 7F–J).

**Figure 8:**
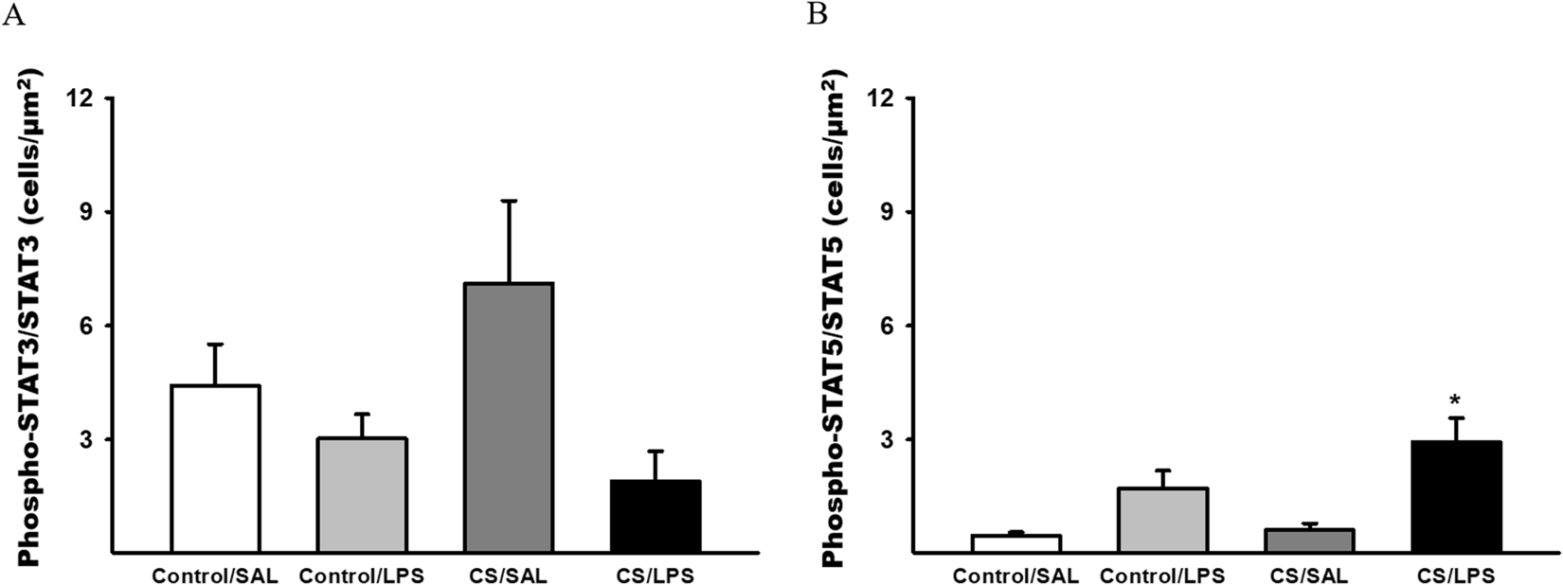
The ratio of normalized of PhosphoStat3/Stat3 and PhosphoStat5/Stat5. (**A**) PhosphoStat3/Stat3, there were no significant differences in parameters values among the experimental groups. Control/SAL n = 7, Control/LPS n = 8, CS/SAL n = 6, CS/LPS n = 3. (**B**) PhosphoStat5/Stat5, *p = 0.002 compared to Control/SAL. Control/SAL n = 9, Control/LPS n = 9, CS/SAL n = 8, CS/LPS n = 4. Kruskal-Wallis, Dunn’s post hoc. Data were expressed as log_10_ base. Data are presented as means and SE.

Regarding the ratio of normalized phosphoSTAT3/STAT3, there were no significant differences among the experimental groups. However, the ratio of phosphoSTAT5/STAT5 was increased in the CS/LPS compared to the Control/SAL and CS/SAL groups (Fig. 8A,B, respectively).

### Measurement of cytokine levels in lung homogenates

**Figure 9:**
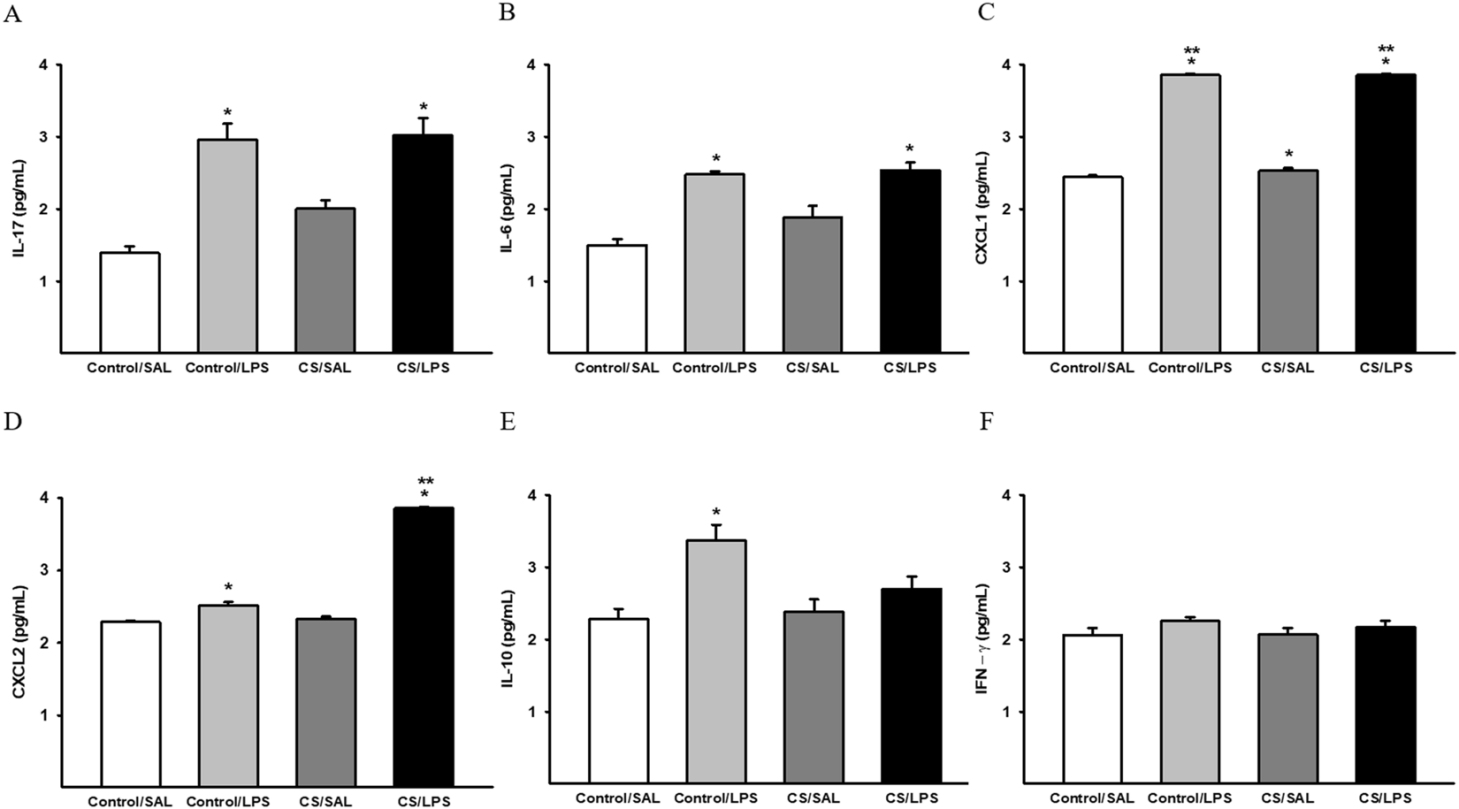
Cytokines and chemokines in lung homogenates. (**A**) IL-17: *p < 0.001 compared to Control/SAL. Control/SAL n = 10, Control/LPS n = 6, CS/SAL n = 8, CS/LPS n = 7. Kruskal-Wallis, Dunn’s post hoc. (**B**) IL-6: *p < 0.001 compared to Control/SAL. Control/SAL n = 10, Control/LPS n = 6, CS/SAL n = 9, CS/LPS n = 7. Kruskal-Wallis, Dunn’s post hoc. (**C**) CXCL1: *p < 0.001 compared to Control/SAL and **p < 0.001 compared to CS/SAL. Control/SAL n = 10, Control/LPS n = 6, CS/SAL n = 9, CS/LPS n = 7. ANOVA, Holm-Sidak post hoc. (**D**) CXCL2: *p < 0.001 compared to Control/SAL and CS/SAL and **p < 0.001 compared to Control/LPS group. Control/SAL n = 10, Control/LPS n = 6, CS/SAL n = 9, CS/LPS n = 7. ANOVA, Holm-Sidak post hoc. (**E**) IL-10: *p < 0.01 compared to Control/SAL and CS/SAL. Control/SAL n = 10, Control/LPS n = 5, CS/SAL n = 7, CS/LPS n = 7. Kruskal-Wallis, Dunn’s post hoc. (**F**) IFN-γ: there were no significant differences in parameters values among the experimental groups. Control/SAL n = 10, Control/LPS n = 6, CS/SAL n = 9, CS/LPS n = 7. Kruskal-Wallis, Dunn’s post hoc. Data were expressed as log_10_ base. Data are presented as means and SE.

The LPS challenge significantly increased IL-17 and IL-6 levels compared to the Control/SAL group (Fig. 9A,B, respectively). In Control/LPS and CS/LPS groups, we also detected increased levels of the CXCL1 and CXCL2 chemokines compared to the Control/SAL and CS/SAL groups. In addition, an increase in CXCL1 levels was detected in the CS/SAL group compared to the Control/SAL group and an increase in CXCL2 levels in the CS/LPS group compared to the Control/LPS group (Fig. 9C,D, respectively). The IL-10 levels were increased in the Control/LPS group compared to the Control/SAL and CS/SAL groups (Fig. 9E). We did not observe significant differences in INF-γ levels among the groups (Fig. 9F).

### Double Staining for Treg and IL-10

**Figure 10:**
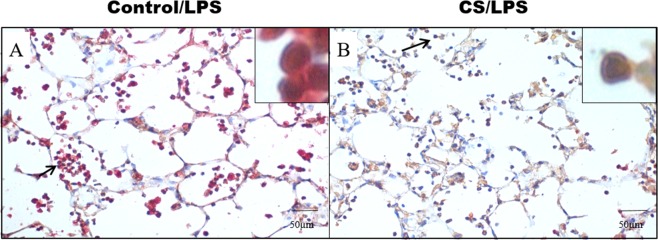
Representative photomicrographs of double immunostaining for FOXP3 (brown reaction product), IL-10 (red reaction product). FOXP3 positive cells was increased in parenchymal areas of CS/LPS while the co-localization of FOXP3/IL-10 (arrow) was found increased in Control/LPS group. (**A**) Control/LPS group and (**B**) CS/LPS group. 400X and 1000X magnification.

The analysis of Treg/IL-10-positive cells revealed an increase in the Control/LPS group compared to the CS/LPS group (Fig. 10A,B, respectively).

## Discussion

CS exposure and subsequent LPS challenge induced an inflammatory process in the BALF and lung parenchyma similar to the process observed in patients with COPD presenting an exacerbation^8,38,39^. Moreover, this dual CS/LPS challenge increased the epithelial thickness and resulted in diffuse alveolar enlargement in the peribronchial and and subpleural airspaces, reflecting a noticeable injury to the parenchymal architecture.

LPS alone increased the densities of both IL-17^+^ cells and Treg cells, but did not increase IL-10 levels in the CS/LPS group. LPS alone also decreased the number of IL-10^+^ cells in this group compared to the Control/LPS group. Therefore, we suggest that decreased IL-10 production plays a pivotal role in the inflammatory exacerbation in this proposed model.

We observed an inflammatory process in the lung parenchyma of CS groups that was mainly characterized by increased numbers of macrophages, neutrophils, CD4^+^ and CD8^+^ T cells, and the subsequent LPS challenge intensified this response. The BALF analysis only revealed statistically significant increases in the numbers of macrophages and total cells in LPS groups. Since the BALF analysis include airways other than the parenchymal areas, an intensified inflammatory response that mainly occurred in parenchymal areas was observed in this animal model.

Alveolar macrophages are known to recruit other cell types to the site of inflammation by releasing chemokines^40^; for example, CXCL1 (chemokine homologous to IL-8) and CXCL2^41^ that are chemoattractants for neutrophils^42^. These findings are in consistent with the higher expression of CXCL1 and CXCL2 in lung homogenates observed in our study.

While CXCL1 expression was increased by exposure to CS or LPS challenge, the levels of CXCL2 were increased only in LPS groups, and the CS/LPS challenge exacerbated this response. Moreover, the administration of both stimuli further increased the neutrophil density in parenchyma, which reinforces the importance of innate immune response in this experimental model^16^.

In patients with COPD, the exacerbation due to bacterial colonization is mainly mediated by IL-8 released by neutrophils^43^, and both neutrophils and IL-8 production are associated with an increase in sputum production and worsening airway obstruction^44–46^.

Regarding T cell subtypes, we observed increased numbers of CD8^+^ and CD4^+^ T cells in both LPS and CS groups, consistent with previous studies using animal models of COPD^31,32,47^; however, we reported a greater increase inin the number of CD4^+^ T cells in animals exposed to CS/LPS. The number of CD8^+^ T cells is increased in the respiratory tract and in the parenchyma of smokers with COPD^48^, and the activation of these cells might contribute to COPD progression^49^.

We analyzed the density of positive cells for STAT3 and phospho-STAT3 and STAT5 and phospho-STAT5 to evaluate the balance between Th17 and Treg differentiation.

Both LPS and CS exposure increased the density of STAT3^+^ cells, and the administration of the combination of CS and LPS exacerbated this increase, corroborating the increase in IL-17 levels and the number of IL17^+^ cells in the lung. Di Stefano and colleagues^50^ showed increased in levels of IL-17A and IL-22 in bronchial mucosal biopsies from patients with stable COPD. In addition, Zhang and colleagues^51^ observed a significant increase in the number of CD4^+^IL17^+^ cells in the alveolar wall of patients with COPD that positively correlated with airway obstruction.

The increase in the number of STAT3^+^ cells is consistent with the increased IL-6 levels in both groups challenged with LPS, since IL-6 is recognized to switch Treg cells to a Th17 response following chronic infection^52^.

On the other hand, the density of phosphoSTAT3^+^ cells was increased in both groups that received LPS, and the administration of CS did not exacerbate this response. Moreover, the numbers of phosphoSTAT3^+^ cells/STAT3^+^ cells were decreased in the CS/LPS group, which was inconsistent with the increased IL-6 and IL-17 levels. Yew-Booth^53^ and colleagues have previously described an increase in STAT3 levels, but not phospoSTAT3 levels, in patients with COPD compared to smokers using imunohistochemsitry, whereas when the authors analyzed the levels of these cytokines using western blotting, they detected increased levels of both STAT3 and phosphoSTAT3. The authors suggested that the antibody used in the study was more suitable for western blotting than for imunhohistochemistry. Although we did not use the same antibody described by Yew-Booth and colleagues^53^, our results are consistent with the previous findings described above, reinforcing the hypothesis that the immunohistochemistry might not be the best technique to evaluate STAT-3 phosphorylation.

Interestingly, we also observed an increase in the density of STAT5^+^ and phosho-STAT5^+^ cells in the LPS groups, and the higher values for phosho-STAT5^+^ cells were observed in the CS/LPS group, corroborating the increased density of Treg cellsand the increased numbers of phosphoSTAT5^+^ cells/STAT5^+^ cells.

Nonetheless, the increase in Treg cells does not reflect an increase in IL-10 release^54^. In the present study, although we observed an increase in the number of Treg cells in both groups that received the LPS instillation, only the Control/LPS group showed concomitantly increased IL-10 levels and IL-10^+^ cells density compared to the other experimental groups.

The increased numbers of other cell types, such as macrophages and neutrophils, in CS/LPS groups that are mediated by the release of this anti-inflammatory interleukin^55^ are insufficient to induce an increase in IL-10 levels.

In the majority of infections, the IL-10 is an essential regulator to control the inflammatory response, and has a more important role than any other cytokine^55^. In viral infections, the IL-10 produced by Treg cells is closely related to the maintenance of the immunopathological balance^56–59^. Nevertheless, the inhibition of IL-10 signaling may exacerbate a pro-inflammatory response that clears pathogens but damages the lung tissue^60^. Jin and colleagues^61^ previously reported similar results in serum from patients with COPD collected during an exacerbation to our findings. The authors showed an increase in IL-17 levels compared to healthy nonsmokers and patients with stable COPD, with a concomitant increase in the number of Treg/CD4^+^ cells. Therefore, although the Tregs are upregulated during acute exacerbations, their generation and differentiation were not sufficient, suggesting that both pro-inflammatory and anti-inflammatory reactions are enhanced, with pro-inflammatory and anti-inflammatory reactions are enhanced, with pro-inflammatory reactions predominating during acute exacerbations in patients with COPD. Additionally, the authors showed normal Treg/IL-17 numbers; however, the Treg cells were not sufficient to suppress the exacerbated inflammatory process.In the present study, we did not evaluate how Treg function impaired IL-10 release. However, we showed the role of the TH17/Treg cytokine imbalance, highlighting the importance of a lack of IL-10 release in this COPD exacerbation model.

## Conclusions

We showed that the Th17/Treg cytokine imbalance lead the inflammatory process exacerbation as well as the diffuse structural changes in lungs in this COPD exacerbation model.

## Data Availability

The datasets generated during and/or analyzed during the current study are available from the corresponding author on reasonable request.
